# Analysis of gene expression profile of peripheral blood in alveolar and cystic echinococcosis

**DOI:** 10.3389/fcimb.2022.913393

**Published:** 2022-08-11

**Authors:** Lei Liu, Fan Chen, Shan Jiang, Bo Zhong, Wei Li, Kejun Xu, Qi Wang, Ying Wang, Jianping Cao

**Affiliations:** ^1^ Department of Hospital Infection Management, Sichuan Provincial Orthopedic Hospital, Chengdu, China; ^2^ National Institute of Parasitic Diseases, Chinese Center for Disease Control and Prevention, (Chinese Center for Tropical Diseases Research); Key Laboratory of Parasite and Vector Biology, National Health Commission of People’s Republic of China; National Center for International Research on Tropical Diseases, China; World Health Organization Collaborating Center for Tropical Diseases, Shanghai, China; ^3^ Department of Microbiological Laboratory, Xindu County Center for Disease Control and Prevention, Chengdu, China; ^4^ Department of Department of Environmental and School Health, Chengdu Center for Disease Control and Prevention, Chengdu, China; ^5^ Institute of Parasitic Diseases, Sichuan Center for Disease Control and Prevention, Chengdu, China; ^6^ Department of Parasitic Diseases, Garzê Center for Disease Control and Prevention, Kangding, China; ^7^ The School of Global Health, Chinese Center for Tropical Diseases Research, Shanghai Jiao Tong University School of Medicine, Shanghai, China

**Keywords:** echinococcosis, peripheral blood, RNA-sequencing, mRNA, DEG

## Abstract

RNA-sequencing (RNA-seq) is a versatile, high-throughput technology that is being widely employed for screening differentially expressed genes (DEGs) in various diseases. Echinococcosis, a globally distributed zoonosis, has been reported to impose a heavy disease burden in pastoral areas of China. Herein we aimed to explore the molecular mechanisms underlying echinococcosis. In this study, peripheral blood samples were collected from six patients with alveolar echinococcosis (AE), six patients with cystic echinococcosis (CE), and six healthy controls. RNA-Seq (mRNA) was performed to detect gene transcript and expression levels, and DEGs were subjected to bioinformatic analyses. In comparison with healthy controls, 492 DEGs (270 upregulated, 222 downregulated) were found in the AE group and 424 DEGs (170 upregulated, 254 downregulated) were found in the CE group (|log_2_ (fold change)| > 1 and P < 0.05). Further, 60 genes were upregulated and 39 were downregulated in both the AE and CE groups. Gene ontology enrichment analysis indicated that DEGs were mainly involved in molecular functions, including extracellular space, extracellular region, organ and system development, and anatomical structure development. Protein–protein interaction (PPI) networks were constructed to depict the complex relationship between DEGs and interacting proteins.

## Introduction

Echinococcosis is one of the most geographically widespread parasitic zoonoses across the world. *Via* peroral infection by the eggs of *Echinococcus* spp., the parasitic metacestode (larval stage) develops as a tumor-like tissue in the liver of intermediate hosts, which include livestock, small rodents, and even humans. In humans, *E. multilocularis* and *E. granulosus* cause alveolar echinococcosis (AE) and cystic echinococcosis (CE), respectively (Kammerer and Schantz, 1993; WHO, 2011; Deplazes et al., 2017). In endemic regions, echinococcosis is a significant public health issue, imposing financial burden on patients due to high treatment and hospitalization costs (Craig et al., 2019; Wang et al., 2020). Therefore, many earlier studies on echinococcosis have focused on exploring disease pathogenesis, diagnostics, and epidemiology, aiding the development of treatment and prevention strategies (Moro and Schantz, 2006; Nunnari et al., 2012; Fu et al., 2020).

At present, echinococcosis diagnosis primarily depends on clinical symptoms, epidemiological contact history and imaging examinations. However, the sensitivity of imaging examinations to detect small lesions at the early stage is not particularly high. Serological antibody detection tests could be used to overcome this gap, but they lack species specificity due to the possibility of cross-reaction with other helminthic antigens (Schweiger et al., 2012).

Surgery is the most commonly used method to treat echinococcosis, but recurrence is an unavoidable issue. Albendazole is the preferred drug recommended by the World Health Organization, which selectively inhibits glucose uptake, leading to glycogen storage depletion in the parasite. However, the efficacy of albendazole is not yet well documented, and it can also cause side effects, such as dizziness, nausea, and leucopenia (Dehkordi et al., 2019). Immunoprophylaxis is a potential satisfactory way to prevent the prevalence of echinococcosis. Several antigens have been evaluated as vaccine candidates, but to date, they remain in the animal experiment stage (Jazouli et al., 2020; Gharibi et al., 2021).

In recent years, with the genome mapping of *Echinococcus* as well as of intermediate and terminal hosts, studies have moved their focus to the genetic level, especially research into gene expression and regulation. Rapid advancements are being made in gene-related technologies, and RNA-sequencing (RNA-seq) based on second generation high-throughput sequencing platforms is receiving a great deal of attention. Transcriptomics, which is widely employed for analyzing gene expression, splicing sites, allele-specific expression, and isomers, has become the mainstream method for gene annotation and expression determination.

In this study, we collected peripheral blood samples from patients with AE, CE and healthy controls, and assessed gene transcripts and expression levels using RNA-Seq technology. The differential expression levels of mRNA between patients and controls were analyzed with bioinformatics. Our findings provide new ideas and clues for echinococcosis diagnosis and treatment; further, we believe they could also support research into immunity.

## Materials and methods

### Study participants

The present study was approved by the Ethics Committee of Sichuan Centre for Disease Control and Prevention (reference no. 2018–2), and all methods were performed in accordance with the relevant guidelines and regulations of the Declaration of Helsinki. All participants were Tibetan residents living in Garzê County, Garzê Tibetan Autonomous Prefecture; their signed written informed consent was obtained before the study. Peripheral blood samples were collected from six patients with AE, six patients with CE, and six healthy controls; all the three groups were included with the same half-and-half sex ratio. None of the study participants showed history of cardiovascular and cerebrovascular diseases, metabolic diseases, tumors, or systemic autoimmune diseases.

### Blood sample collection and RNA extraction

From each participant, 4 mL venous blood were drawn into an ethylenediaminetetraacetic acid vacutainer tube and diluted with an equal volume of sterile phosphate-buffered saline. Total RNA was isolated using TRIzol; the TRIzol:blood ratio was 3:1. RNA quality was assessed using the RNA Nano 6000 Assay Kit of the Bioanalyzer 2100 system (Agilent Technologies, CA, USA).

### RNA preparation, library construction, and illumina sequencing

A total amount of 3 µg RNA per sample was used as input material for RNA sample preparations. Sequencing libraries were generated using NEBNext^®^ Ultra™ RNA Library Prep Kit for Illumina^®^ (NEB, USA), according to manufacturer instructions, and index codes were added to attribute sequences to each sample. mRNA was purified from total RNA using poly-T oligo-attached magnetic beads. Fragmentation was achieved using divalent cations under elevated temperature in NEBNext^®^ First Strand Synthesis Reaction Buffer (5×). First-strand cDNA was synthesized using random hexamer primers and M-MuLV reverse transcriptase (RNase H-), and second-strand cDNA was synthesized using DNA polymerase I and RNase H. cDNA fragments of preferentially 250–300 bp in length were selected for PCR, and library quality was assessed on an Agilent Bioanalyzer 2100. The libraries were sequenced on an Illumina HiSeq platform and 125 bp/150 bp paired-end reads were generated. Raw data were cleaned with Cutadapt 4.0 to remove reads that were of low quality and those containing adapter and poly-N.

### Read mapping and gene expression quantification

Reference genome and gene model annotation files were downloaded from the genome website Ensembl (http://www.ensembl.org/index.html). An index of the reference genome was built using Bowtie v2.2.3, and paired-end clean reads were aligned to the reference genome using TopHat v2.0.12. HTSeq v0.6.1 was used to count the number of reads mapped to each gene. Subsequently, the expected number of fragments per kilobase of transcript sequence per millions base pairs (FPKM) was calculated for estimating gene expression levels (Anders et al., 2015).

### Differential expression analysis

Differential expression analysis was performed using the DESeq R package (1.18.0), which provided statistical methods for determining differential expression in digital gene expression data using a model based on negative binomial distribution. Differentially expressed genes (DEGs) were identified by comparing the expression levels of all transcripts in the AE and CE groups with those in the control group. Genes with |log2 (fold change)| > 1 and an adjusted P-value <0.05 were considered to be differentially expressed (Anders and Huber, 2010; Anders and Huber, 2016).

### Enrichment analysis

Gene ontology (GO) enrichment analyses of DEGs were performed using the GOseq R package, in which gene length bias was corrected. GO terms with corrected P < 0.05 were considered significantly enriched by DEGs (Young et al., 2010).

ClusterProfiler R package was used to assess the statistical enrichment of DEGs in KEGG pathways and to analyze their involvement in pertinent metabolic pathways.

### Protein–protein interaction network

PPI networks were constructed using Cytoscape v3.7.2, and Search Tool for the Retrieval of Interacting Genes (STRING) database was employed to depict the complex relationship between DEGs and proteins encoded by them (Szklarczyk et al., 2015).

### Verification of the expression of DEGs by quantitative RT-PCR

To verify the accuracy of our sequencing results, qRT-PCR was performed to investigate the expression levels of several DEGs. The mRNA was reverse-transcribed into cDNA using the Revert Aid First-Strand cDNA Synthesis Kit (Thermo, USA). The real-time PCR was performed using SYBR Premix Ex Taq II (Takara, Japan). GAPDH served as the internal reference gene, and primers were designed using Primer Premier 5. Each PCR was repeated in triplicate. The expression levels of genes were normalized by 18S rRNA and analyzed using the 2^−△△Ct^ method.

## Results

### Differential expression analysis

**Figure 1:**
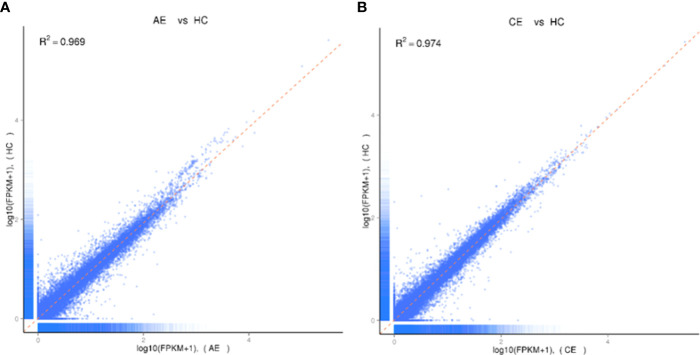
Analysis of homogeneity between AE, CE, and healthy control samples. **(A)** Expression analysis of AE and healthy control samples. The Pearson correlation coefficient was 0.969. **(B)** Expression analysis of CE and healthy control samples. The Pearson correlation coefficient was 0.974.

A total of 1.02 billion raw reads were obtained, and the average number of reads per sample was 56,609,372. The error rates of 18 specimens were all less than 0.03%. Bases with Q_Phred_ ≥ 20 accounted for 96.85% of the total number. Correlation analyses showed that the average global profiles of gene expressions between AE, CE, and control samples were highly correlated (correlation coefficient R^2^_AE_ = 0.995, R^2^_CE_ = 0.994, **Figure 1**).

**Figure 2:**
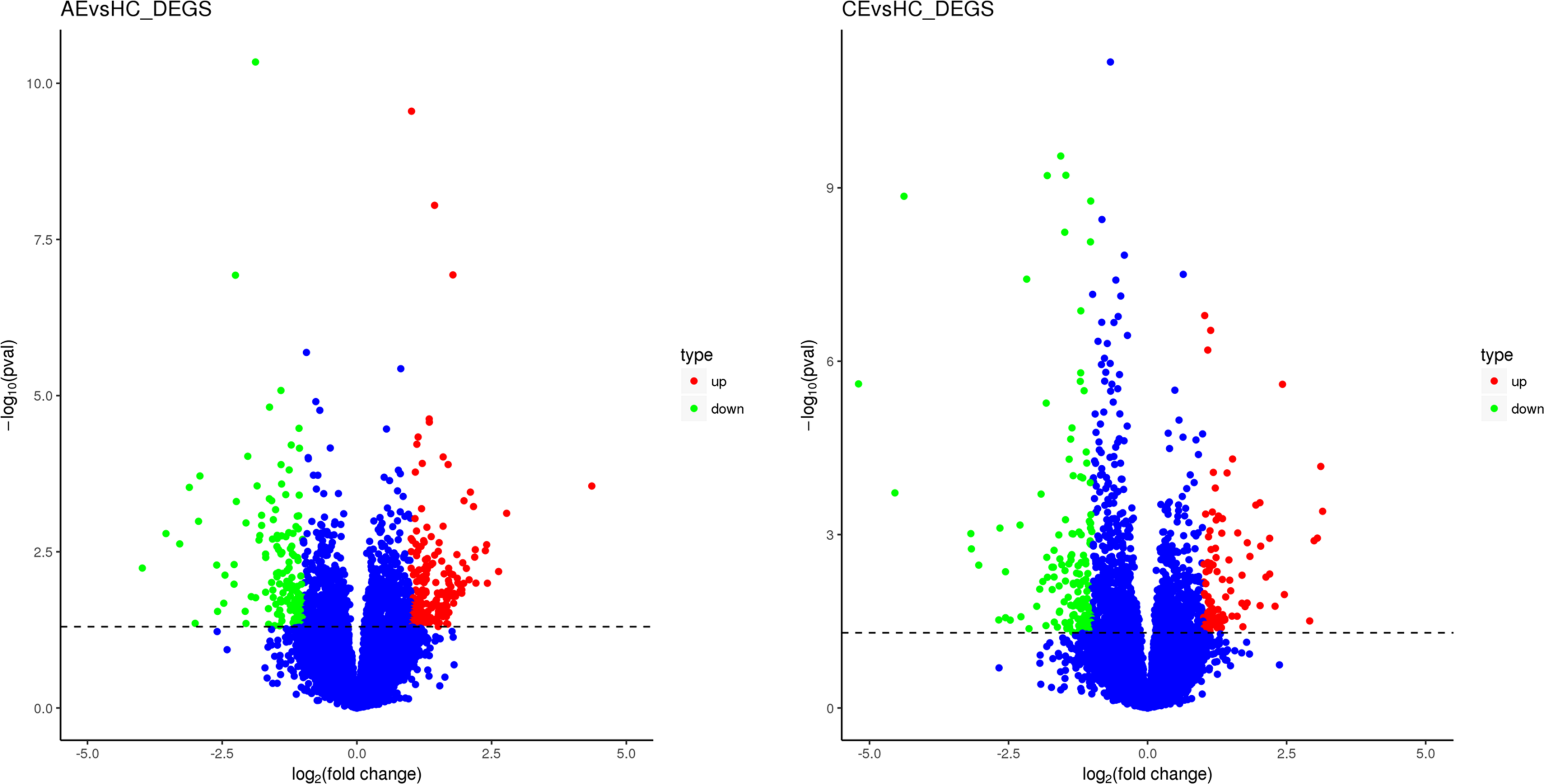
Scatterplot of DEGs. **(A)** The expression levels of 270 and 222 genes were up- and downregulated, respectively, in patients with AE. **(B)** The expression levels of 170 and 254 were up- and downregulated, respectively, in patients with CE.

**Figure 3:**
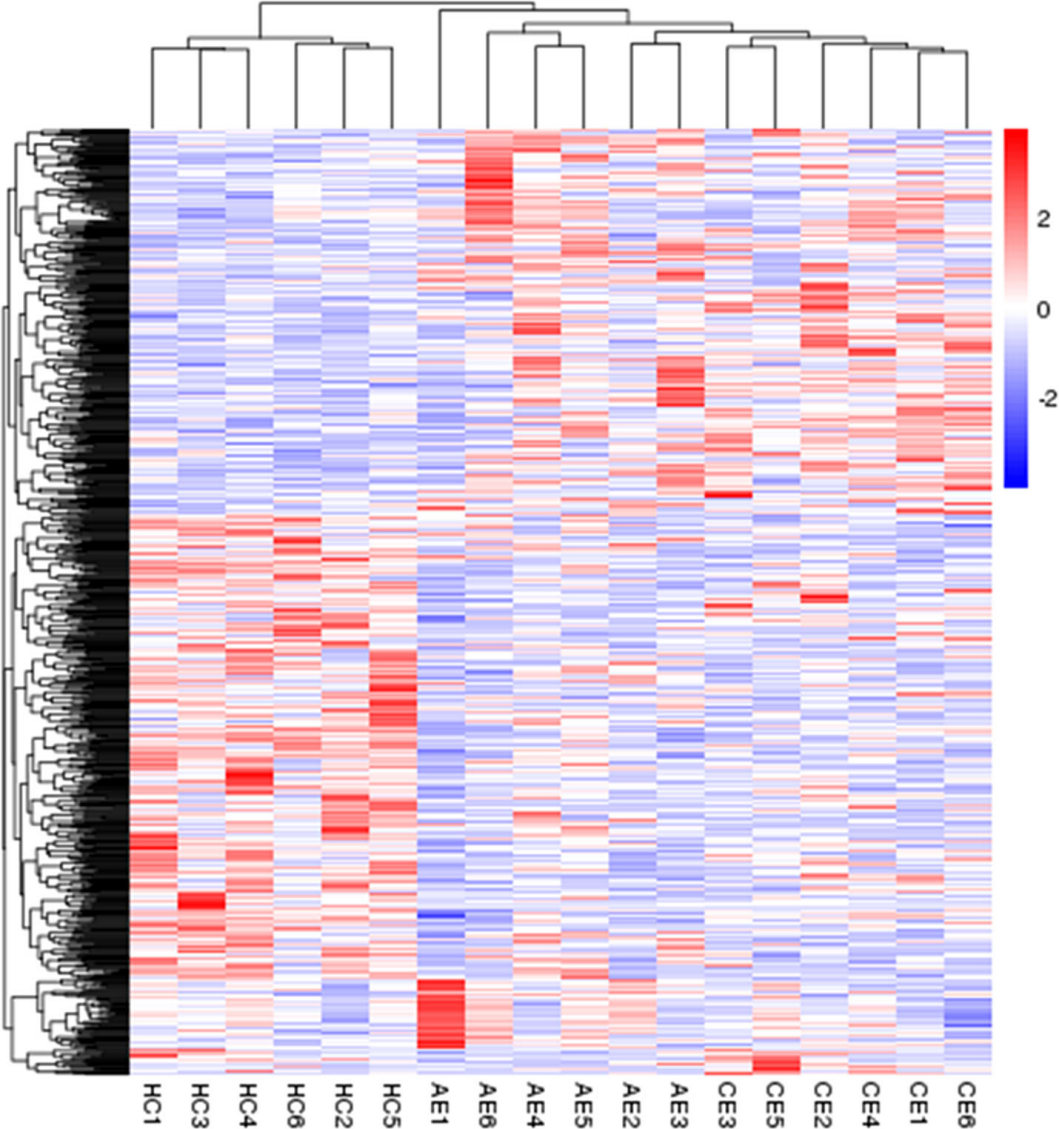
Heatmap of DEGs. The heatmap was generated using normalized FPKM values of DEGs. The expression level of each transcript is represented by a color, ranging from blue (low) to red (high).

In comparison with the control group, 492 (270 upregulated, 222 downregulated) and 424 (170 upregulated, 254 downregulated) DEGs were identified in the AE and CE groups, respectively (**Figure 2**). Volcano plots were drawn using log_2_ (fold change) as the abscissa and −log_10_ (*t*-test significance P-value) as the ordinate. Hierarchical cluster analysis was performed to assess DEG expression patterns, which became evident *via* a heatmap (**Figure 3**).

**Figure 4:**
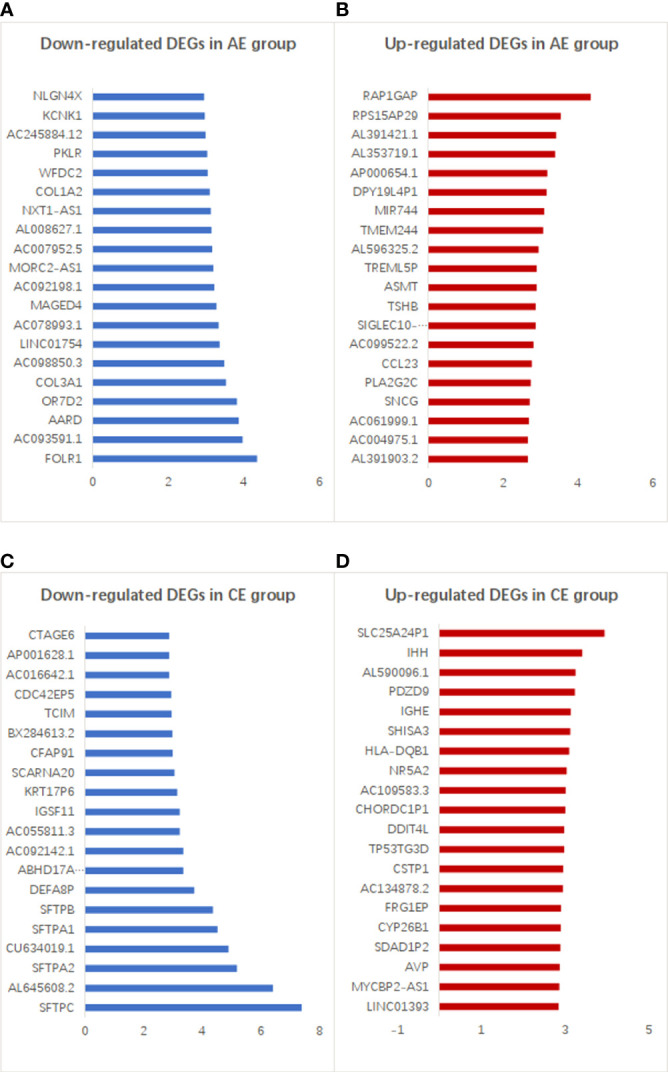
Top 20 up- and downregulated DEGs in the AE and CE groups. **(A)** Down- and **(B)** upregulated genes with higher differential expression levels in patients with AE. **(C)** Down- and **(D)** upregulated genes with higher differential expression levels in patients with CE.

The top 20 downregulated and upregulated DEGs in the AE and CE groups are shown in **Figure 4**. The expression levels of some genes were found to be upregulated (e.g., RAP1GAP, RPS15AP29, and AL391421.1) and downregulated (e.g., FOLR1, AC093591.1, and AARD) in patients with AE. Similarly, the expression levels of many genes were upregulated (e.g., SLC25A24P1, IHH, and AL590096.1) and downregulated (e.g., SFTPC, AL645608.2, and SFTPA2) in patients with CE.

**Figure 5:**
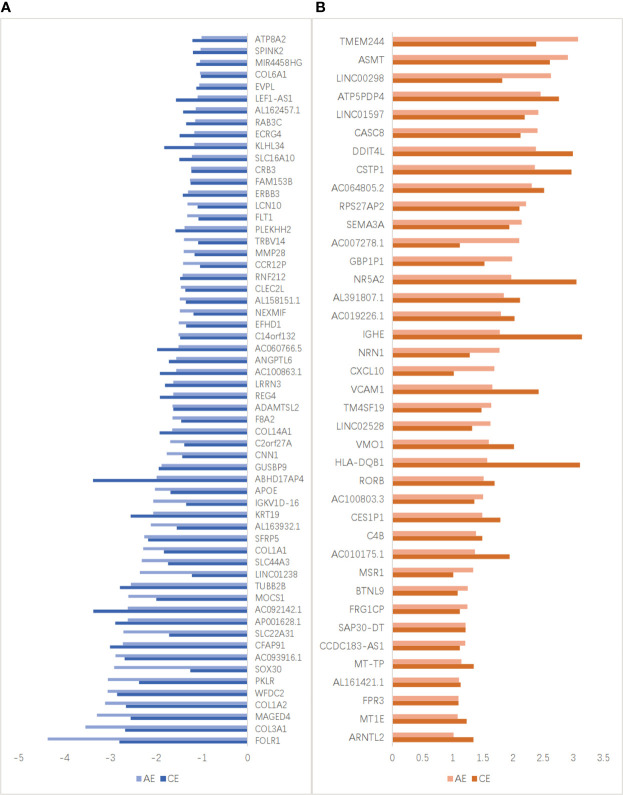
DEGs detected both in the AE and CE groups. **(A)** The expression levels of 39 genes, including FOLR1, COL3A1, and MAGED4, were downregulated both in the AE and CE groups. Light blue and dark blue lines represent the FPKM of downregulated DEGs in patients with AE and CE, respectively. **(B)** The expression levels of 60 genes, including TMEM244, ASMT, and LINC00298, were upregulated both in the AE and CE groups. Light red and dark red lines represent the FPKM of upregulated DEGs in patients with AE and CE, respectively.

AE and CE are pathologically different but also show some similarities. The expression of some genes was up- or downregulated in both AE and CE (**Figure 5**). The expression levels of 60 genes (e.g., ASMT, and TMEM244) were upregulated, and those of 39 genes (e.g., FOLR1, COL3A1, and MAGED4) were downregulated in both these groups.

### GO enrichment analysis

**Figure 6:**
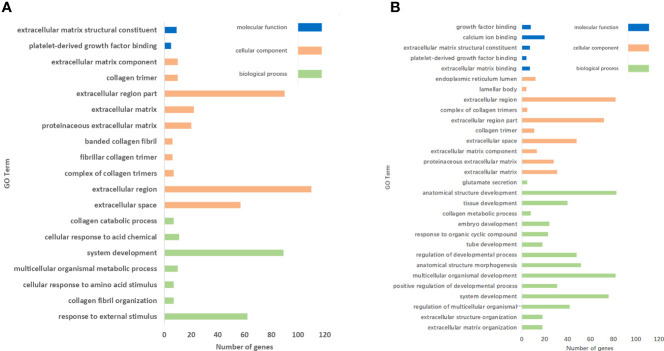
GO enrichment analysis of DEGs. **(A)** Nineteen and **(B)** 30 clusters were significantly annotated in the AE group and the CE group, respectively.

In the AE group, 19 clusters (*P* < 0.05) were significantly annotated: 7, 10, and 2 were associated with the functional categories of biological process, cellular component, and molecular function, respectively (**Figure 6A**). The top enrichments were closely related to extracellular space, extracellular region, organ and system development, and response to external stimulus.

In the CE group, 30 clusters (*P* < 0.05) were significantly annotated: 15, 10, and 5 were associated with the functional categories of biological process, cellular component, and molecular function, respectively (**Figure 6B**). The top upregulated enrichments were closely related to extracellular space, extracellular region, anatomical structure development, multicellular organismal development, and system development.

### KEGG pathway enrichment analysis

**Figure 7:**
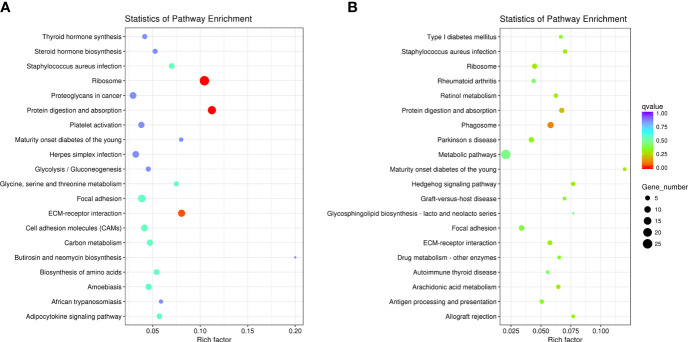
KEGG pathway enrichment analysis of DEGs. **(A)** In the AE group, DEGs were significantly involved in 11 pathways, and **(B)** in the CE group, DEGs were significantly involved in 16 pathways.

In the AE group, DEGs were mainly involved in 11 pathways (P < 0.05), including ribosome, protein digestion and absorption, and ECM-receptor interaction (**Figure 7A**). In the CE group, DEGs were mainly involved in 16 pathways (*P* < 0.05), including phagosome and protein digestion and absorption (**Figure 7B**).

### PPI network analysis

**Figure 8:**
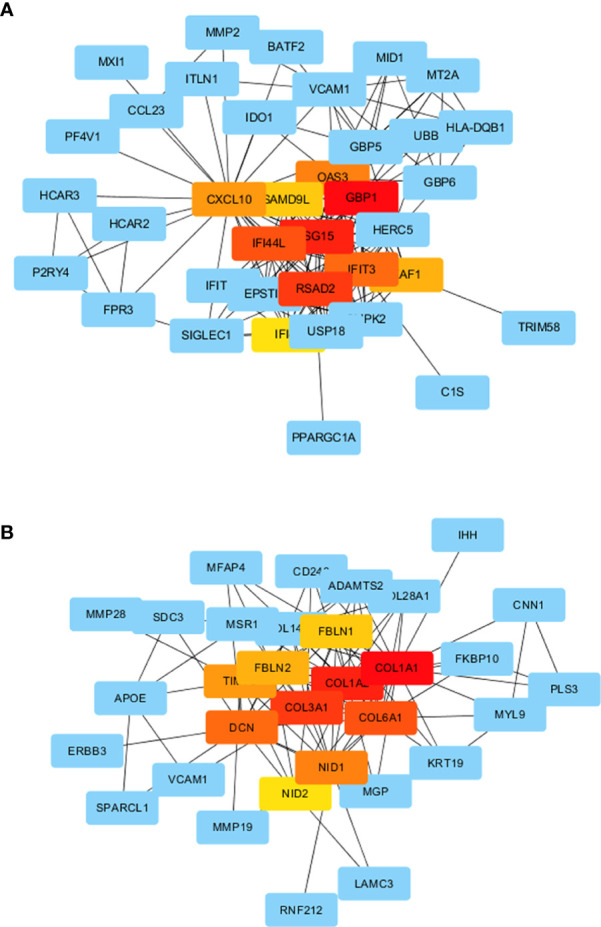
PPI networks. **(A)** PPI network for the AE group and **(B)** CE group.

Proteins encoded by DEGs in the AE and CE groups formed a complex PPI network (**Figure 8A, B**). The PPI network of protein products in the AE group was mainly linked by hub genes such as GBP1, ISG15, RSAD2, CXCL10, and VCAM-1, while that of those in the CE group was mainly linked by hub genes such as COL1A1, COL1A2, COL3A1, DCN, and FBLN1.

### Verification of the expression of DEGs by qRT-PCR

**Figure 9:**
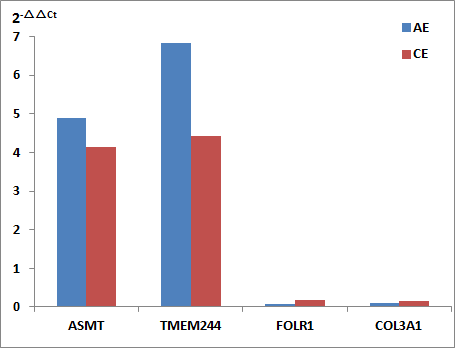
Expression levels of DEGs by qRT-PCR. Relative expression levels (2^−△△Ct^) of ASMT, TMEM244, FOLR1, and COL3A1 in the AE and CE groups.

qRT-PCR was performed to validate the expression levels of four DEGs: ASMT, TMEM244, FOLR1, and COL3A1. The primer sequences were described in **Table 1**. The relative expression levels of ASMT and TMEM244 were found to be upregulated, whereas those of FOLR1 and COL3A1 were downregulated in the AE and CE groups. The results of the 2^−△△Ct^ method were consistent with those of RNA-seq (**Figure 9**).

**Table 1:** Primer sequences used in the qRT-PCR experiment.

Gene	Forward sequence	Reverse sequence
GAPDH	CTCCTCCACCTTTGACGCT	TCTTCCTCTTGTGCTCTTGCT
ASMT	GCATGTCTCTGTACCCTGGA	GGTCTTTGAAGAAATCCCCTTCC
TMEM244	GCGTGATGTTTGAGGTGCAT	CCCAAACCCATTCTTCCACAAC
FOLR1	CCCTGGAGGAAGAATGCCTG	CCAGCTCTGATCCACCTGCT
COL3A1	TTGGGTTGTCTAATATGGT	TCTCAGGATTTGTAGGGAT

GAPDH, Glyceraldehyde-3-phosphate dehydrogenase; ASMT, Acetylserotonin O-methyltransferase; TMEM244, Transmembrane protein 244; FOLR1, Folate receptor alpha; COL3A1, Collagen alpha-1(III) chain.

## Discussion

High-throughput sequencing technology has been widely applied to identify reliable molecular markers and to elucidate mechanisms underlying different diseases. Herein we conducted a comprehensive transcriptomic study that involved healthy controls and patients with AE and CE, and detected several DEGs by RNA-Seq. In subsequent analyses, we specifically focused on DEGs that showed considerably aberrant expression levels and those that were key nodes in the PPI networks.

The expression level of *guanylate-binding protein 1* (*GBP1*) gene, encoding guanylate-binding protein 1 (GBP1) belonging to the dynamin family, was upregulated in patients with AE and CE. GBP1 specifically binds to guanine nucleotides, and its expression is induced by interferon (IFN)-γ and other inflammatory factors, such as IFN-α, IFN-β, IL-1α, IL-1β, and TNF-α. In addition to its association with inflammation, GBP1 participates in cell-autonomous defense against intracellular pathogens. Moreover, in recent years, GBP1 has been reported to play a pivotal role in the occurrence and development of tumors, including liver cancer. Its expression was found to be positively correlated with disease severity and tissue differentiation degree (Takagi et al., 2011). In patients with HCV, IFN induces the upregulation of the expression of GBP1, which binds to NS5B polymerase, inhibiting viral replication (Tretina et al., 2019). GBP1 was also demonstrated to be a key protective factor against *Toxoplasma gondii* infection in human mesenchymal stem cells. Its recruitment to the parasitophorous vacuole membrane in IFN-γ–stimulated human mesenchymal stem cells was identified to significantly inhibit *T. gondii* replication (Qin et al., 2017). Further, pathological staining of liver specimens of patients with echinococcosis showed infiltration of macrophages, neutrophils, eosinophils, and T lymphocytes around lesions (Díaz et al., 2016), causing atypical protein expression.

The expression level of *C-X-C motif chemokine 10* (*CXCL10*) gene, encoding C-X-C motif chemokine 10 (CXCL10), was upregulated in patients with AE and CE. CXCL10 is secreted by several types of cells, including monocytes, endothelial cells, and fibroblasts, in response to IFN-γ. CXCL10 binds to the chemokine receptor CXCR3, which is expressed by Th1 cells, and induces lymphocytes to migrate to the infection site, causing lymphatic infiltration and local immune response. Meanwhile, the recruited Th1 cells secrete IFN-γ, which stimulates the local production of CXCL10 (Booth et al., 2002; Colvin et al., 2004). Serum CXCL10 levels in chronic HBV patients have been reported to be significantly higher than those in healthy controls and to be positively correlated with serum ALT and HBV DNA levels. Besides, serum CXCL10 levels in patients with HBC were also found to be associated with the severity of tissue necrotizing inflammation, with the increase in CXCL10 levels serving as an independent influencing factor for predicting the degree of fibrosis (Wang et al., 2008; You et al., 2011; Grebely et al., 2013). Higher serum CXCL10 levels may recruit more CD4^+^ T cells for metastasis to the liver, leading to a more severe immune response; echinococcosis might have a similar mechanism.

The expression level of *vascular cell adhesion molecule-1* (*VCAM-1*) gene, encoding vascular cell adhesion molecule-1 (VCAM-1), was upregulated in patients with AE and CE. VCAM-1 is an inducible cell surface sialoglycoprotein that was first discovered on cytokine-activated endothelium. It is expressed in large and small blood vessels after cytokine stimulation, and mediates adhesion of lymphocytes, monocytes, eosinophils, and basophils to vascular endothelium. Once endothelial cells are stimulated by cytokines, VCAM-1 is released into the bloodstream following proteolytic cleavage by vascular endothelial activation mediated by proinflammatory cytokines (Pirisi et al., 1996; Wang et al., 2014). VCAM-1 has been suggested to play a role in the pathogenesis of chronic hepatitis and cirrhosis, as its expression levels were found to be upregulated in chronic liver disease (Antonova et al., 2013; Wadkin et al., 2017; Ali et al., 2020). Furthermore, VCAM-1 levels were observed to be significantly higher in HBsAg-positive patients and positively correlated with ALT. VCAM-1 is evidently involved in the destruction of liver cells as it participates in the killing process mediated by T and NK cells and in antibody-dependent cellular cytotoxicity mediated by neutrophils and monocytes.

*Collagen 1A1 (COL1A1)* and *COL1A2* genes encode type I collagen α1 and α 2, respectively; *COL3A1* gene encodes type III collagen (Exposito et al., 2010). In this study, the expression levels of these genes were downregulated in the AE and CE groups. Collagens are a family of proteins that strengthen and support many tissues, including the cartilage, bone, tendon, skin, and sclera (Liu et al., 2016; Augusciak-Duma et al., 2018). Collagen fibers constitute the main component of the extracellular matrix (ECM). Their excessive synthesis and reduced degradation can accordingly cause abnormal proliferation of fibrous connective tissue and promote fibrosis development, which is one of the important clinical manifestations of echinococcosis in the liver. COL1A1, COL1A2, and COL3A1 have been previously reported to be abnormally overexpressed in some tumors and parasitic diseases (Li et al., 2016; Tao et al., 2018; Ma et al., 2019). Mice infected with echinococcosis were also found to show higher COL1A1 and COL3A1 expression levels in the hepatic tissue (Wang et al., 2012). In this study, the expression levels of these genes in the blood samples were different. Some connection might exist between these contradictory results, and thus, further studies are warranted.

The expression level of *fibulin-1*(*FBLN1*) gene, which encodes fibulin-1, was significantly upregulated in patients with CE, but not in those with AE. As a member of the fibulin glycoprotein family, fibulin-1 is an ECM and blood glycoprotein, and it functions as a bridge in the organization of ECM supramolecular structures. Fibulin-1 can also be incorporated into fibronectin-containing matrix fibers to play a role in cell adhesion and migration along protein fibers within the ECM. It is implicated in cellular transformation and tumor invasion, and it can behave both as an oncosuppressor and oncogene depending on the tissue environment (de Vega et al., 2009; Obaya et al., 2012). In a recent study, FBLN1 expression was found to be significantly downregulated in exosomes derived from the plasma of patients with HIV, and was particularly altered in HIV^+^ smokers (Kodidela et al., 2020). Similar results were reported for serum exosomal fibulin-1 in patients with gastric cancer (Yang et al., 2016). Some other studies have also reported that fibulin-1 expression is significantly upregulated in some cancers, validating its role in promoting tumor occurrence and development (Moll et al., 2002; Bardin et al., 2005). The changes in FBLN1 levels are related to pathological characteristics, but the underlying mechanism appears to be complex and thus warrants an in-depth investigation.

In this study, the application of RNA-Seq generated high quality, accurate data. RNA expression patterns observed in this study indicated that many immunological factors encoded by DEGs might be involved in the pathogenesis of echinococcosis. The results indicate possible target genes related to the pathogenesis of echinococcosis through statistical tests. The top DEGs identified herein could serve as potential diagnostic biomarkers and a basis for further biological and functional investigations.

This study also had a limitation. The sample size was small, which might not provide sufficient evidence to support our hypotheses. Further studies with a larger sample size are thus warranted to identify even more DEGs and to investigate their relationship with echinococcosis more comprehensively.

## Conclusions

DEGs with the most significant differences in expression levels and hub genes in PPI networks might serve as potential diagnostic biomarkers. Further, they also can serve as a basis for biological and functional investigations of echinococcosis with larger sample sizes.

## Data availability statement

The datasets analyzed for this study can be found in the Genome Sequence Archive for Human (GSA-Human, the National Genomics Data Center), https://bigd.big.ac.cn/gsa-human/browse/HRA001670.

## Ethics statement

The present study was approved by the Ethics Committee of Sichuan Centre for Disease Control and Prevention (reference no. 2018–2), and all methods were performed in accordance with the relevant guidelines and regulations of the Declaration of Helsinki.

## Author contributions

Conceived and designed the experiments: LL, JC, BZ, and WL. Collected the samples: FC, QW, and KX. Performed the experiments: LL, FC, SJ, and QW. Analyzed the data: LL, FC, SJ, and YW. Wrote the paper: LL, JC, and YW. All authors have read and approved the manuscript.

## Funding

The research was supported by the Comprehensive Prevention and Control Pilot Project on Echinococcosis in Shiqu County, the China Postdoctoral Science Foundation (No. 2019M650800 to LL), the National Nature Science Foundation of China (No. 81971969 to JC), and the Three-Year Public Health Action Plan (2020-2022) of Shanghai (No. GWV-10.1-XK13 to JC).

## Acknowledgments

We would like to thank the staff at the Garzê County Center for Disease Control and Prevention (Garzê Prefecture, Sichuan Province) for their assistance with volunteer recruitment and sample collection.

## Conflict of interest

The authors declare that the research was conducted in the absence of any commercial or financial relationships that could be construed as a potential conflict of interest.

## Publisher’s note

All claims expressed in this article are solely those of the authors and do not necessarily represent those of their affiliated organizations, or those of the publisher, the editors and the reviewers. Any product that may be evaluated in this article, or claim that may be made by its manufacturer, is not guaranteed or endorsed by the publisher.
